# Validation of proteins associated with pathological damage in human tuberculosis granulomas: study protocol

**DOI:** 10.12688/wellcomeopenres.19226.1

**Published:** 2023-03-28

**Authors:** Thabo Mpotje, Jessica More, Kerishka Rajkumar-Bhugeloo, Denelle Moodley, Mohlopheni J Marakalala

**Affiliations:** 1 School of Laboratory Medicine and Medical Sciences, University of KwaZulu-Natal, Durban, KwaZulu-Natal, 4001, South Africa; 2 Basic and Translational Science, Africa Health Research Institute, Durban, KwaZulu-Natal, 4001, South Africa; 3 Division of Infection and Immunity, University College London, London, England, UK

**Keywords:** Mycobacterium tuberculosis, TB Granulomas, Host-Directed Therapies, Inflammation, Lung pathology

## Abstract

The presence of the Tuberculosis (TB) disease-causing pathogen, *Mycobacterium Tuberculosis* (Mtb), induces the development of a pathological feature termed granuloma, which the host uses to contain the bacteria. However, the granuloma may dissociate resulting in detrimental caseation of the lung. The disease contributes to a growing global burden of lung function challenges, warranting for more understanding of the TB-induced immunopathology. The current study aims to explore in detail host factors that drive pathological features of TB contributing to extensive lung tissue destruction. Lung tissue sections obtained from patients undergoing surgical resection will be processed and analyzed using histopathological assays including Immunohistochemistry, Immunofluorescence, Hematoxylin and Eosin staining and Laser Capture Microdissection. The findings will provide key host factors that associate with exacerbated lung immunopathology during TB.

## Background

Although the current treatment of TB is effective at curing the disease, most survivors develop some form of persisting pulmonary dysfunction ( Hnizdo & Murray 1998). This dysfunction can cause minor abnormalities to severe breathlessness which potentiates the risk of death from other potential pulmonary complications ( Pasipanodya *et al.*, 2010; Ralph *et al.*, 2013; Schünemann *et al.*, 2000). Patients who recover from TB have been reported to contribute greatly to an increasing global burden of obstructive pulmonary diseases ( Amaral *et al.*, 2015; Byrne *et al.*, 2015). Furthermore, the development of TB-induced granulomatous pathology in the affected tissue adds to reduced efficacy of drugs leading to prolonged treatment period ( Datta *et al.*, 2015; Kokesch-Himmelreich *et al.*, 2022). More work is required to fully understand the tissue immunopathology during TB, and current gaps in the knowledge presents a challenge in fully unravelling mechanisms that can be utilized to develop effective therapies against the disease. 

**Figure 1.**
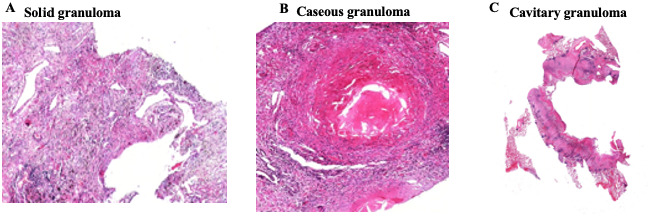
Representative images of solid ( **A**), caseous ( **B**) and cavitary tuberculosis granulomas. Adapted from Marakalala *et al.*, 2016.

This study aims to explore in detail the pathological features of TB such as caseation, which may lead to extensive lung tissue destruction ( Hunter, 2011). Caseation is predominantly known to be driven by excessive inflammatory responses mediated by myeloid cells, including macrophages and neutrophils ( Muefong & Sutherland, 2020). The myeloid cells are initially recruited to the site of infection leading to formation of a granuloma to help neutralize or contain the invading bacteria ( Ndlovu & Marakalala, 2016). From the influence of both pathogen and host factors, the granuloma can undergo complex remodeling events leading to formation of a variety of granulomas ranging from a more protective solid granuloma to severe caseous and cavitary granuloma ( Figure 1) ( Ndlovu & Marakalala, 2016; Silva Miranda *et al.*, 2012).

A previous study from our group has identified proteomic signatures that were differentially expressed across the varying regions of the TB-induced granulomas ( Marakalala *et al.*, 2016). In the current study we will utilize histopathological assays to explore in detail, host factors that are associated with exacerbated granulomatous pathology in TB-disease participants which were identified from the previous study ( Marakalala *et al.*, 2016).

## Methods

### Study setting and tissue sample collection

Lung tissue sections/samples (n=14) were obtained from TB patients undergoing therapeutic resection for advanced TB-associated lung damage, at a collaborating hospital called King Dinizulu Hospital Complex based in Durban, South Africa. The study, which is part of an ongoing lung study program at the Africa Health Research Institute, was approved by the Biomedical Research Ethics Committee (BREC) at the University of KwaZulu-Natal (BE019/13). Personal identifying data was allocated to each patient giving a written consent. Following lung resection, a variety of tissue sections ranging from non-affected to mild and severe TB-induced immunopathology, were carefully isolated and preserved in 4% Formaldehyde solution.

### Tissue sample processing

***Removal of formalin from fixed tissue and preparation of slides.*** The lung tissue sections will be processed using Microm STP 120 Spin Tissue Processor (Thermo Scientific, USA) according to the manufacturer’s instruction to remove the 4% Formaldehyde solution from the tissues. Following this, the tissue sections will be embedded into wax blocks using HistoStar Embedding Station which dispenses heated paraffin in a liquid form. It will then be solidified on a cold center. For preparation of paraffin slides, tissue samples on a wax block will be cut at 5μM sections and mounted on sialinized slides (Plus slides) and then melted at 65°C for 2 hours.

***Paraffin removal from embedded tissue.*** The slides will be dipped into xylene three times for five minutes each to wash the paraffin. This will be followed by rehydration of the tissue samples by dipping and incubating the slides into absolute ethanol (100%), then 95% ethanol, and finally 70% ethanol with each incubated for 10 minutes (2x 5-minute intervals) for each of the solutions. The slides will then be transferred to Tris buffer solution and soaked for 1 hour. The endogenous peroxidase will be quenched by dipping the slides into aqueous solution of 3% peroxidase for three minutes. The slides will then be rinsed with Tris buffer for three minutes.

***Antigen retrieval.*** To retrieve the antigens on the tissue sections, the slides will be placed into a pressure cooker using full racks to hold the slides (for empty spaces place blank glass slides). The top and lid of the pressure cooker will then be assembled and locked into position. The cooker will be placed into a 700 – 900Watt microwave oven and cooked on high power for 40 minutes. Following the cooking step, the steam will be released by removing the rubber gasket from the pressure cooker. Once the steam is completely released, the lid will be opened, and the slides carefully removed. Lastly, the slides will be placed in Tris/saline buffer.

### Tissue staining

***Hematoxylin and Eosin (H&E) staining.*** The H&E staining will be carried out on formalin-fixed paraffin embedded slides prior to paraffin removal. The slides will be dipped into xylene two times for five minutes each to wash off the paraffin. This will be followed by rehydration of the tissue samples by dipping the slides into absolute ethanol (100%) for ten minutes (2x 5 minutes intervals), then 95% ethanol for ten minutes (2x 5 minutes intervals), and finally 70% ethanol for ten minutes (2x five minutes intervals). The slides will then be transferred to Tris buffer solution and soaked for one hour. The slides will be dipped in hematoxylin for five minutes, then washed in tap water for five minutes. Following the wash step, the slides will then be dipped into Eosin for two minutes, and then rinsed in 95% ethanol for 30 seconds. This will be followed by washing in absolute ethanol two times for one minute. The slides will then be rinsed in xylene for one minute. A mounting media will be added to the tissue on the slides and covered with coverslips.

***Immunohistochemistry (IHC) staining.*** Tissue sample staining: For blocking, 1% goat serum will be added to the tissue sections for 20 minutes to block non-specific immunostaining. The primary antibody of interest will then be added in an appropriate concentration that is recommended by the manufacturer or previous literature, and then incubated for 1 hour at room temperature (RT). The antibody will be washed from the slides using Tris buffer and the sections soaked in Tris buffer for 10 minutes (2x 5 min washes). The slides will then be incubated with corresponding biotinylated goat anti‐species antibody for 10 minutes. This will then be followed by rinsing the slides using Tris buffer for 10 minutes (2x 5 minutes washes). The detection enzyme solution (streptavidin peroxidase) will then be added, and the slides incubated in a dark humid chamber for 5 minutes. The slides will be rinsed in Tris buffer for 10 minutes (2x 5 minutes washes). A solution of chromogen, 3,3′‐diaminobenzidine (DAB) at a concentration of 1mg/ml in Tris buffer with 0.016% fresh hydrogen peroxide will be added to the slides and incubated for 8 minutes. The DAB will then be washed from the slides using tap water for 1 minute.

Addition of counter staining: The slides will be dipped into a solution of hematoxylin (or Methyl Green) that is diluted 1:1 in distilled water, and then incubated for 1 minute to produce a very light nuclear counterstain. The slides will be washed using double distilled water, and then dehydrated by dipping 95% ethanol for 1 minute, then 100% ethanol for 1 minute. The slides will then be washed 3 times using xylene and a coverslip mounted on the slides. The slides will be scanned using Hamamatsu nanozoomer 2.0RS scanner (ThermoScientific, USA) to identify the staining of interest.

***Immunofluorescence (IF) staining.*** For multicolor Immunofluorescence staining of lung tissues following antigen retrieval, the slides will be washed using 1x EnVision ^TM^ Flex wash buffer (DM831, ref.: K8000/K8002, Agilent, USA) for 10 minutes (2x 5 minutes washes). A hydrophobic pen will then be applied around the tissue of interest to allow ease of staining. Endogenous peroxidase activity on the tissue will be blocked using peroxidase-blocking buffer (S202386-2, Agilent, USA) for 10 minutes at RT using peroxidase-blocking buffer (S202386-2, Agilent, USA), and then washed for 10 minutes (2x 5 minutes washes) using the wash buffer. A second blocking buffer (0.05g BSA + 0.5ml (10%) Goat serum + 4.5ml wash buffer) will then be applied for 20 minutes to ensure non-specific binding is limited. Following this, the slides will be washed for 10 minutes (2x 5 minutes washes) using the wash buffer. The primary antibody of interest at a concentration recommended by manufacturer or previous literature, will then be added to the slides and incubated for 45 minutes at RT. Following incubation, the slides will be washed as above for ten minutes (2x 5 minutes washes) using the wash buffer. A secondary antibody will then be applied by adding three drops of Opal Polymer (HRP: Ms + Rb) to the slides for 20 minutes to be conjugated to the coated primary antibody. For the bound antibodies to be detected, a fluorochrome of specific wavelength 494/525nm (OPAL 520 reagent, AKOYA Biosciences, USA) will be added to the slides at a concentration recommended by the manufacturer (i.e. 1/200 dilution in 1x Plus Amplification buffer) and incubated for ten minutes. To add another specific antibody of interest, the tissue sections on the slides will be subjected to antigen retrieval using TSC solution (10% of Antigen Retrieval 6 buffer (K800421-2, Agilent, USA) diluted in distilled water). The slides will be microwave heated in three steps; the first will be to run the microwave set on high for two minutes, followed by low for five minutes, and then on medium for ten minutes. Once done the slides will then be subjected to cooling for 20 minutes at RT using tap water. To equilibrate the tissue, the slides will be rinsed in 1x EnVision ^TM^ Flex wash buffer (DM831, ref.: K8000/K8002, Agilent, USA) for five minutes at RT. A blocking buffer (0.05g BSA + 0.5ml (10%) will then be added to the slides and incubated for ten minutes to block non-specific binding. The second primary antibody at a concentration recommended by the manufacturer will be added and the slides incubated overnight. The following day, the slides will be washed, and an Opal polymer secondary antibody added, followed by addition of a second fluorochrome of a different wavelength, 550/570nm (OPAL 570 reagent, AKOYA Biosciences, USA) as before. A second antigen retrieval will be performed to allow for the addition of a third primary antibody together with an Opal polymer secondary antibody, and a third fluorochrome of a different wavelength, 676/694nm (OPAL 570 reagent, AKOYA Biosciences, USA) following the steps described above. A counterstain, prepared by adding one drop of DAPI into 1ml of wash buffer, will be added to the slides and incubated for 5 minutes at RT. The slides will then be washed in wash buffer as before, then three drops of Dako fluorescent mounting media will be added to the tissue, and coverslips carefully placed over the tissue.

### Laser capture microdissection (LCM)

For the LCM, tissue embedded sections will be cut at 8μM sections and mounted on RNAse free Membrane Slides (cat.: 7950102, Molecular Machines & Industries, USA). The tissue embedded membrane slides will then be placed on the center stage of the Infrared (IR) laser and inverted light microscope. The tissue of interest will then be visualized to identify the region of interest on the tissue. The laser control tower will be used to focus and highlight/draw around the region of interest. The IR laser will then be activated to allow removal of the region of interest from the heterogeneous tissue section. The system will then pick up the dissected region and place it onto Isolation Caps Transparent 0.5ml (cat.: 7950204 MMI, Molecular Machines & Industries, USA). The collected sample will then be sent for transcriptomic analysis or MS-proteomics. 

## Discussion

There is a considerable growth in the global burden of lung dysfunction as a result of TB ( Amaral *et al.*, 2015; Byrne *et al.*, 2015). Furthermore, the development of tissue immunopathology also contributes to reduced access by currently available TB drugs into the site of infection ( Datta *et al.*, 2015; Kokesch-Himmelreich *et al.*, 2022). It is, therefore, crucial to understand the mechanisms and host factors that associate with the development of tissue immunopathology to improve the therapeutic strategies aimed at alleviating or controlling pulmonary impairment resulting from TB disease. The work aims to evaluate host factors that associate with TB-induced lung immunopathology as potential targets for host-directed therapy (HDT) development.

HDTs are a promising tool to combat disease complications such as TB, as they can be used to exploit host factors that modulate or drive specific pathways with crucial roles in disease pathogenesis ( Palucci & Delogu, 2018). In terms of TB disease progression, a previous study has identified host proteins that associated with varying forms of granulomas ( Marakalala *et al.*, 2016). Some of these proteins were driving key inflammatory pathways that contribute to TB disease progression ( Marakalala *et al.*, 2016). The histopathological assays from this proposed study will be used to characterize key host proteins which serve as potential targets for the development of HDTs against TB disease. 

Lung tissue samples with varying degrees of damage ranging from non-affected to moderate and finally severe TB-induced immunopathology, will be characterized to identify multiple granuloma types including solid, caseous and cavitary granulomas. The non-affected region will be used as an alternate control to healthy lung tissue, as the latter cannot be obtained. Although the tissue sections originate from the individuals suffering from TB, the differences between non-affected and TB-induced immunopathological sections have been demonstrated to provide enough details to delineate host factors that associate with the disease immunopathogenesis ( Guler *et al.*, 2021; Marakalala *et al.*, 2016).

Histopathological assays presented in the current protocol will be useful in confirming and delineating tissue distribution of key inflammatory and or regulatory host proteins of interest in healthy and TB diseased organ. This will provide strong evidence to suggest key host factors as targets of HDTs or biomarkers of TB disease progression.

## Study status

Lung tissue samples from patients with written consent have been collected, and tissue embedded slides have also been prepared. Tissue sections with proven TB-induced immunopathology as determined using H&E, have been identified. From this, several inflammatory host factors have been demonstrated to associate with exacerbated tissue pathology as demonstrated using IHC and IF.

## Data Availability

No data are associated with this article.
