# Supplementary material for: Validation of proteins associated with pathological damage in human tuberculosis granulomas: study protocol
Source: Wellcome Open Res. 2023 Mar 28;8:139. [Version 1] doi: 10.12688/wellcomeopenres.19226.1 (PMC10113799; doi:10.12688/wellcomeopenres.19226.1)
Supplement: Peer review history

# Peer review: Reviewer response for version 1

**DOI:** 10.21956/wellcomeopenres.21309.r55881

**Published:** 2023-04-18

**Authors:** Gabriel Mashabela

**Affiliations:** 1 SAMRC/NHLS/UCT Molecular Mycobacteriology Research Unit, Department of Pathology and Institute of Infectious Disease and Molecular Medicine, Faculty of Health Sciences, DSI/NRF Centre of Excellence for Biomedical TB Research, University of Cape Town, Rondebosch, Western Cape, South Africa

Granuloma is one of the most important features of TB and unraveling all factors (host and pathogen-derived) contributing to its dynamism may hold a key towards better control and treatment of TB disease. This study combines visual and omics techniques to discern host factors contributing to extensive granulomatous pathology in participants with TB disease.

**Comments**

 The study concept is very good and the protocol is well written. However, observed minor errors are brought to the attention of the authors:

 (1) In the *Methods* section: Formaldehyde should be written in small caps.

 (2) Under *Paraffin removal from embedded tissue* subsection: "3% peroxidase" should be replaced by "3% hydrogen peroxide". 

 (3) Under *Immunofluorescence (IF) staining* subsection: the line "using peroxidase-blocking buffer (S202386-2, Agilent, USA)" was repeated.

 (4) In the *Methods* section: the should be a space between a number and unit matrix (e.g., 0.5 ml instead of 0.5ml). This should be corrected in most parts of the *Methods* section.

 (5) Although this not an error, per se, but more towards consistence, there was extensive use of full word for numbers, e.g., ten minutes especially under subsection *Immunofluorescence (IF) staining*. This seems to be in contrast to most sections of the *Methods* sections where number (e.g., 10 minutes) were used.

 (6) Under *Immunohistochemistry (IHC) staining* subsection, the line of the paragraph: I presume "and incubated"  should be added before "for 20 minutes"?

 Lastly, an additional statement in the *Background* such as "The work aims to evaluate host factors that associate with TB-induced lung immunopathology as potential targets for host-directed therapy (HDT) development", which was mentioned in the *Discussion* section, will be useful in providing a strong motivation for conducting a study like this.

Is the study design appropriate for the research question?

Yes

Is the rationale for, and objectives of, the study clearly described?

Yes

Are sufficient details of the methods provided to allow replication by others?

Yes

Are the datasets clearly presented in a useable and accessible format?

Yes

Reviewer Expertise:

Biochemistry, Molecular Biology, Drug Discovery

I confirm that I have read this submission and believe that I have an appropriate level of expertise to confirm that it is of an acceptable scientific standard.

# Peer review: Reviewer response for version 1

**DOI:** 10.21956/wellcomeopenres.21309.r55880

**Published:** 2023-04-13

**Authors:** Ngiambudulu M. Francisco

**Affiliations:** 1 Grupo de Investigação Microbiana e Imunológica, Instituto Nacional de Investigação em Saúde (National Institute for Health Research), Luanda, Angola

I am happy that such protocol on "Validation of proteins associated with pathological damage in human tuberculosis granulomas: study protocol" has been designed.

 Tuberculosis (TB) remains a major global health problem, and there are so many mechanisms that are still scientifically unexplained. Granunoma compartment is complex, this study is going to set light on how pathological damages in the granulomas of individuals with TB occur.

**Major Comments:**

 Although a mention has been made in background section as follows “…In the current study we will utilize histopathological assays to explore in detail, host factors that are associated with exacerbated granulomatous pathology in TB-disease participants which were identified from the previous study (Marakalala *et al., *2016)...", a similar explanatory mention should be added in the method section.

 The authors should give a brief objective, purpose(s) or reason(s) of each tissue staining techniques mentioned in the protocol. Readers would like to know why the authors want to perform these types of staining. The referred techniques are mentioned below:

- Hematoxylin and Eosin (H&E) staining.
- Immunohistochemistry (IHC) staining.
- Immunofluorescence (IF) staining.
- Laser capture microdissection (LCM).

**Other comments:**

 Overall, the protocol is well written.

Is the study design appropriate for the research question?

Yes

Is the rationale for, and objectives of, the study clearly described?

Yes

Are sufficient details of the methods provided to allow replication by others?

Yes

Are the datasets clearly presented in a useable and accessible format?

Yes

Reviewer Expertise:

Pathogen immunomodulation and Genomic epidemiology.

I confirm that I have read this submission and believe that I have an appropriate level of expertise to confirm that it is of an acceptable scientific standard.
